# Line-Field Confocal Optical Coherence Tomography May Enhance Monitoring of Superficial Basal Cell Carcinoma Treated with Imiquimod 5% Cream: A Pilot Study

**DOI:** 10.3390/cancers13194913

**Published:** 2021-09-30

**Authors:** Anna Elisa Verzì, Giuseppe Micali, Francesco Lacarrubba

**Affiliations:** Dermatology Clinic, University of Catania, 95123 Catania, Italy; aeverzi@gmail.com (A.E.V.); giuseppe.micali@unict.it (G.M.)

**Keywords:** line-field confocal optical coherence tomography, LC-OCT, basal cell carcinoma, imiquimod, treatment monitoring

## Abstract

**Simple Summary:**

Line-field confocal optical coherence tomography (LC-OCT) is a new non-invasive technique that allows the visualization of the epidermis and dermis and their different structures and findings at the cellular level, providing a sort of “virtual biopsy”. The interest of using LC-OCT in the diagnosis of skin disorders is growing. The purpose of this study was to investigate if LC-OCT may be useful to enhance the monitoring of superficial basal cell carcinoma (BCC) in a series of patients treated with IQ 5% cream, an immune response modifier currently approved in Europe and the USA. In our experience, LC-OCT was able to show sub-clinical signs of BCC after treatment, leading to a further cycle of IQ 5% cream. Based on our preliminary results, LC-OCT may represent a promising tool able to enhance the evaluation of the treatment response of BCCs to non-surgical treatments.

**Abstract:**

Line-field confocal optical coherence tomography (LC-OCT) is a novel, non-invasive technique for real-time skin imaging. Imiquimod (IQ) 5% cream is an immune response modifier currently approved for the treatment of small, superficial basal cell carcinoma (BCC). The aim of this study was to investigate if LC-OCT may be useful to enhance the treatment monitoring of BCC. Twenty superficial BCCs from 12 patients were treated with IQ 5% cream once daily, five days a week, for six weeks. Clinical and LC-OCT evaluations were performed at baseline and 4 weeks after the end of treatment. At the end of the study, 13 lesions showed a complete clinical and LC-OCT response, 4 lesions a partial clinical and LC-OCT response, and 3 lesions a complete clinical response but residual tumoral signs at LC-OCT. Our pilot study suggests that LC-OCT may represent a promising tool able to enhance the evaluation of the treatment response of BCCs to non-invasive treatments. In our case series, its use highlighted, through a detailed, fast, and complete examination of the treated area, three cases of residual BCC that otherwise would have gone undetected at clinical examination. Future studies on larger series of patients treated with different modalities and with a longer follow-up are advisable.

## 1. Introduction

Line-field confocal optical coherence tomography (LC-OCT) is a novel, non-invasive technique for real-time skin imaging [1,2]. It is based on a two-beam interference microscope with a combination of line illumination of the sample using a broadband light source (class one supercontinuum laser with a central wavelength of 800 nm) and line detection (vertical section images from several A-scans acquired sequentially) using a line scan camera (that reads the image data one line at a time). It measures the echo time delay and amplitude of light backscattered from cutaneous microstructures through low-coherence interferometry associated with confocal spatial filtering [1,2].

LC-OCT allows the recognition and measurement of the different layers of the epidermis and dermis and their structures and findings at the cellular level, providing a sort of “virtual biopsy”. It combines the advantages of both reflectance confocal microscopy (RCM) and optical coherence tomography (OCT) in terms of spatial resolution, penetration, and image orientation. In particular, it is able to provide high-resolution images (axial: 1.1 μm; lateral: 1.3 μm) in both vertical (or “en coupe”, similarly to conventional OCT) and horizontal (or “en face”, similarly to RCM) sections, with a penetration depth of up to 500 μm. Therefore, the resolution is higher than for conventional OCT (which is ~10 μm) and the penetration depth is higher than for RCM (which is ~200 μm) [3]. Moreover, LC-OCT allows three-dimensional reconstructions and the acquisition of videos. The real-time acquisition of images is painless and causes no tissue damage; as a result, LC-OCT is totally safe to use on children and pregnant women.

The interest of using LC-OCT in the diagnosis of skin disorders is growing. Reported applications include benign dermal melanocytic proliferations [4], skin tumors such as actinic keratoses, squamous and basal cell carcinoma (BCC) [1,5,6,7,8], sebaceous hyperplasia [9], xanthogranuloma [10], autoimmune bullous diseases [11], herpes infection [12], scabies [13], aquagenic keratoderma [14], Kaposi’s sarcoma [15], and circumscribed palmar hypokeratosis [16].

In particular, in BCC, the main LC-OCT features described include lobulated structures (tumor islands or nests) with variable shape, size, and location within the dermis. Blood vessels within the dermis are also visualized as hypo-reflective structures of various shape and size, with hyperreflective elements flowing within them [1,6]. Other structures frequently observed in BCC are represented by bright cells of various shape and size both within the epidermis and the lobules, histopathologically correlating to immunologically competent skin cells and activated melanocytes [6]. Lobules represent the main criterion as they correspond to the most distinctive histological feature of BCC: the aggregates of basaloid cells into the dermis. Lobules typically reveal an outer bright rim (possibly due to the compression exerted by the tumor islands on the collagen fibers) surrounding a middle dark rim (corresponding to peritumoral mucin deposition) and an inner grey core (corresponding to the dense cellularity) [6]. Because the orientation of the basaloid cells is prevalently parallel to the epidermis, these laminated structures resemble a specific pattern named as “millefeuille”. LC-OCT allows the ability to assess the shape and location of the lobules, which are crucial to ascertain the BCC subtype. In superficial BCC, the lobules are connected to the epidermis. In nodular BCC, the stroma is modified, and the lobules are larger and not connected to the epidermis. In infiltrative BCC, the lobules are branched [6].

Imiquimod (IQ) is an immune response modifier currently approved in Europe and the USA as a 5% cream for the treatment of small, superficial BCCs in immunocompetent adults, applied five times per week for 6 weeks. In several studies it represented a valid alternative to surgery in the treatment of low-risk, single or multiple superficial BCC (grade of recommendation: A), with a complete response in about 80% of cases and good cosmetic outcomes [17,18,19,20].

The purpose of this study was to investigate if LC-OCT may be useful to enhance the monitoring of superficial BCC in a series of patients treated with IQ 5% cream.

## 2. Materials and Methods

Twelve immunocompetent patients affected by single or multiple, primary, and small (<2 cm), superficial BCCs located outside of the high-risk T-zone of the face were enrolled in an open, pilot study. The diagnosis of BCC was based on clinical, dermoscopic, and LC-OCT examinations. Patients with Gorlin’s syndrome and/or immunosuppressed were excluded. The study was approved by the local ethics committee and all subjects gave written informed consent prior to entry.

At baseline (T0), patients were instructed to apply, on one or more BCCs, a thin layer of IQ 5% cream once daily, five consecutive days of the week, for six weeks, according to the leaflet schedule. The treatment area included a 1 cm margin of skin around the tumor. Clinical and LC-OCT evaluations were performed at baseline and 4 weeks after the end of treatment (T1, 10 weeks from baseline) in order to evaluate the response to treatment. During follow-up visits, missed applications were also checked.

LC-OCT was performed with the commercially available DeepLive™ (DAMAE Medical, Paris, France), which provides images with an axial resolution of 1.1 μm, a lateral resolution of 1.3 μm, and a field of view of 1.2 mm × 0.5 mm × 0.5 mm. The device has a central unit connected to a handheld probe and a monitor where images are displayed in a grey scale based on the light backscattering from the sample microstructures. Immersion oil is applied between the glass window of the probe and the skin surface for index matching, thus reducing the specular back reflection [1]. As the operator gently moves the probe over the patient skin, live images are displayed on the monitor in real time. The compact handheld probe has a small imaging head, which is advantageous for examining difficult-to-access regions of the skin (including lips, ears, periocular area, conjunctiva, genitals, glans mucosa, interdigital spaces, etc.). The probe integrates a dermoscopic camera that allows the visualization of the exact area where the examination is performed, also reducing the risks connected to relocation or slipping of the probe.

Characteristic LC-OCT features indicative of superficial BCC included the presence of lobules (characterized by a millefeuille pattern, clefting, and a bright rim) connected to the epidermis.

## 3. Results

A total of 20 superficial BCCs from 12 Caucasian patients (5 males and 7 females, mean age 67.4 years, range 31–82) were evaluated (Table 1). Twelve BCC were located on the trunk, five on the face, one on the neck, one on the shoulder, and one on the forearm.

**Figure 1:**
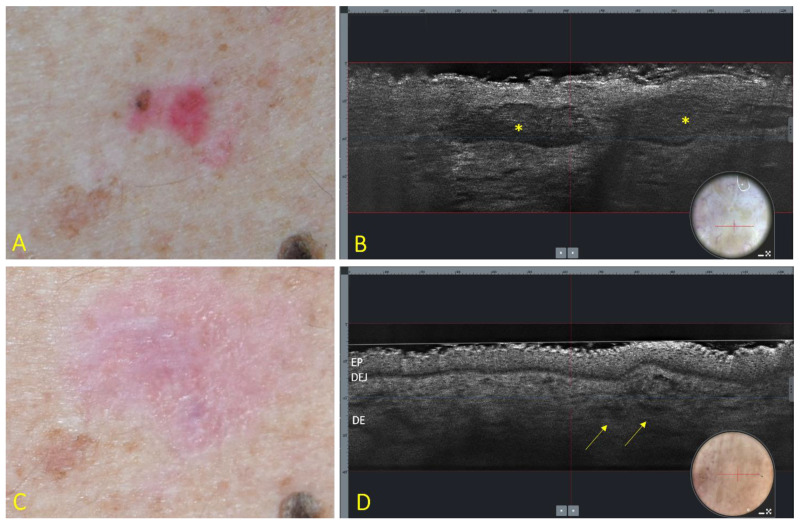
Superficial BCC of the trunk at baseline (**A**,**B**) and 4 weeks after the end of treatment with IQ 5% cream (**C**,**D**). (**A**) Clinical aspect showing a 16 × 14 mm, pink–reddish, and partially pigmented patch. (**B**) LC-OCT vertical section (field of view: 1200 × 250 μm) showing the presence of lobules (asterisks) connected to the epidermis, with an outer bright rim surrounding a middle dark rim, and an inner grey core with a “millefeuille” pattern. Insert: dermoscopy of the examined area (field of view: 1200 × 500 μm); the red line corresponds to the vertical section. (**C**) Clinical disappearance of the lesion with mild residual erythema. (**D**) LC-OCT showing disappearance of the lobules with persistence of dilated vessels (arrows). EP = epidermis; DEJ = dermoepidermal junction; DE = dermis. Insert: dermoscopy of the examined lesion (field of view: 1200 × 500 μm); the red line corresponds to the vertical section.

**Figure 2:**
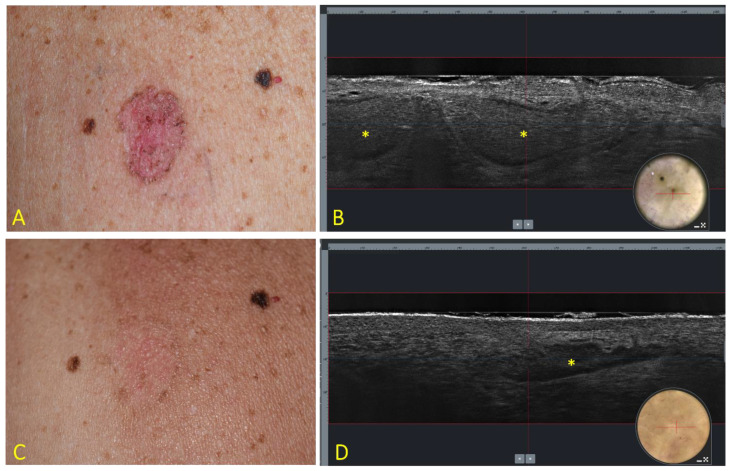
Superficial BCC of the trunk at baseline (**A**,**B**) and 4 weeks after the end of treatment with IQ 5% cream (**C**,**D**). (**A**) Clinical aspect showing a 18 × 12 mm, roundish, and reddish patch with pigmented areas. (**B**) LC-OCT vertical section (field of view: 1200 × 250 μm) showing the presence of lobules (asterisks) connected to the epidermis with an outer bright rim surrounding a middle dark rim, and an inner grey core with a “millefeuille” pattern. Insert: dermoscopy of the examined lesion (field of view: 1200 × 500μm); the red line corresponds to the vertical section. (**C**) Clinical disappearance of the lesion with mild residual erythema. (**D**) LC-OCT showing persistence of BCC lobules (asterisk). Insert: dermoscopy of the examined area (field of view: 1200 × 500μm); the red line corresponds to the vertical section.

At baseline (T0), all lesions showed, at LC-OCT, the presence of the characteristic features indicative of superficial BCC. All patients completed the 6 weeks of treatment, and no missed application was recorded. Nine patients reported the expected side effects of IQ such as erythema, edema, vesicles, erosions, ulcerations, excoriations, exudation, and crusting. After 10 weeks from baseline (T1), 13 lesions showed a complete clinical and LC-OCT response (Figure 1), 4 lesions showed a partial clinical and LC-OCT response and were addressed by surgical excision, and 3 lesions showed a complete clinical response but persistent signs of residual BCC at LC-OCT (lobules connected to the epidermis) (Figure 2), which were addressed by a further cycle of IQ 5% cream.

## 4. Discussion

Basal cell carcinoma (BCC) is the most common non-melanoma skin cancer, with incidence rates rising each year [17]. Although rarely metastasizing, if not recognized and adequately treated it can lead to significant local destruction and morbidity. More than 95% of BCCs are easily treated with standard surgery or medical/physical alternative treatments [21]. Surgical therapy represents the “gold standard” because of high cure rates and histological confirmation of tumor clearance. However, in cases of low-risk BCC, such as small, superficial BCC and small, thin, and nodular BCC in immunocompetent subjects, some nonsurgical treatment options may be considered, including IQ 5% cream, 5-fluorouracil 5%, and photodynamic therapy [20,22].

The aim of this pilot study was to assess if LC-OCT may non-invasively enhance the treatment monitoring of BCC. In general, the response of BCC to non-surgical treatment is assessed by clinical inspection which, however, cannot exclude the persistence of subclinical disease responsible for relapses. On the other hand, post-treatment biopsies for histopathology confirmation of resolution are rarely performed, as they only analyze partial samples. Additionally, multiple biopsies impair the advantages of a lack of invasiveness and good aesthetic results due to the use of non-invasive treatments [23]. Based on this, the use of non-invasive techniques able to recognize sub-clinical signs of BCC persistence is advisable, and some experiences have been published utilizing dermoscopy, reflectance confocal microscopy, and optical coherence tomography [23,24,25,26,27,28,29,30].

Our pilot study suggests that LC-OCT may represent a promising tool that is able to enhance the evaluation of the treatment response of BCCs to non-invasive treatments, such as IQ 5% cream. In our case series, its use highlighted, through a detailed, fast, and complete examination of the treated area, three cases of residual BCC that otherwise would have gone undetected at clinical examination. In particular, after completing the 6-week treatment cycle with IQ 5% cream, clinical examination showed, in agreement with the existing literature, a complete response in 80% of cases (16 out of 20 BCC) and a partial response in the remaining 20% (four cases). However, LC-OCT analysis revealed residual BCC lobules in 7 out of 20 lesions, with three of these lesions appearing to have cleared at clinical evaluation. All lesions presenting clinical and/or LC-OCT persistence of BCC were addressed by surgical excision or by a further cycle of IQ 5% cream. Of note, the integrated dermoscopic camera allowed a precise, timely positioning over the same areas of interest before and after treatment.

In conclusion, our data show that LC-OCT may reveal microscopic signs of residual disease in some superficial BCCs treated with IQ 5% cream that appear clinically healed, thus suggesting further treatment. Limitations of our study include the small sample size, the lack of biopsy before and after treatment, and the short follow-up period. Another limit is the lack of resolution of LC-OCT at deeper layers, so the deep presence of BCC nests in the dermis after treatment cannot be excluded, although in the case of superficial BCC this occurrence seems quite rare [23,29]. Future studies on larger series of patients treated with different modalities and with a longer follow-up are advisable.

## Figures and Tables

**Table 1:** Summary of results.

Patient’s Number	Sex/Age (Years)	Localization	Lesion Size (mm)	Response after 10 Weeks (Four Weeks after the End of Treatment)	
				Clinical	LC-OCT
Pt. 1	F/43	Chest	14 × 9	Complete	Complete
		Back	20 × 16	Complete	Partial
		Shoulder	10 × 8	Complete	Complete
Pt. 2	F/72	Chest	12 × 12	Complete	Complete
		Chest	18 × 10	Complete	Complete
Pt. 3	F/66	Back	18 × 16	Complete	Complete
Pt. 4	M/73	Back	20 × 20	Complete	Partial
		Back	16 × 14	Complete	Complete
		Back	18 × 15	Complete	Complete
Pt. 5	F/79	Back	16 × 5	Complete	Partial
Pt. 6	M/79	Neck	12 × 5	Complete	Complete
Pt. 7	M/82	Right cheek	20 × 18	Partial	Partial
		Right cheek	18 × 13	Complete	Complete
		Left cheek	20 × 20	Partial	Partial
		Left cheek	18 × 18	Complete	Complete
Pt. 8	M/73	Back	16 × 14	Complete	Complete
Pt. 9	M/69	Back	20 × 13	Complete	Complete
Pt. 10	F/65	Chest	20 × 17	Partial	Partial
Pt. 11	F/77	Forearm	18 × 17	Complete	Complete
Pt. 12	F/31	Right cheek	18 × 16	Partial	Partial

## Data Availability

The data presented in this study are available on request from the corresponding author.
